# Increase in Dickkopf-1 Serum Level in Recent Spondyloarthritis. Data from the DESIR Cohort

**DOI:** 10.1371/journal.pone.0134974

**Published:** 2015-08-27

**Authors:** Gaetane Nocturne, Stephan Pavy, Saida Boudaoud, Raphaèle Seror, Philippe Goupille, Philippe Chanson, Désirée van der Heijde, Floris van Gaalen, Francis Berenbaum, Xavier Mariette, Karine Briot, Antoine Feydy, Pascal Claudepierre, Philippe Dieudé, Joanne Nithitham, Kimberly E. Taylor, Lindsey A. Criswell, Maxime Dougados, Christian Roux, Corinne Miceli-Richard

**Affiliations:** 1 Institut Pour la Santé et la Recherche Médicale (INSERM) U1184, Université Paris-Sud 11, Le kremlin Bicêtre, France; 2 Service de rhumatologie, Assistance Publique-Hôpitaux de Paris (AP-HP), Hôpital Bicêtre, Le Kremlin Bicêtre, France; 3 Service de rhumatologie, CHU, Tours, France; UMR CNRS 7292, Université François Rabelais, Tours, France; CIC-INSERM 1415, Tours, France; 4 Service d’endocrinologie, Assistance Publique-Hôpitaux de Paris (AP-HP), Hôpital Bicêtre, Le Kremlin Bicêtre, France; 5 Leiden University Medical Center, Leiden, The Netherlands; 6 Department of Rheumatology and Internal Medicine, LUMC, Leiden, The Netherlands; 7 Sorbonne Universités, UPMC University Paris 6, AP-HP, Hôpital Saint-Antoine, Rheumatology Department, Paris, France; 8 Service de Rhumatologie B, Assistance Publique-Hôpitaux de Paris (AP-HP); Université Paris-Descartes, Paris, France; 9 Service de radiologie, Hôpital Cochin, Assistance Publique-Hôpitaux de Paris (APHP), Paris, France; 10 Service de Rhumatologie, Hôpital Henri-Mondor, Assistance Publique-Hôpitaux de Paris (APHP), Créteil, France; 11 Service de Rhumatologie, Hôpital Bichat, AP-HP, Paris, France; 12 Rosalind Russell / Ephraim P Engleman Rheumatology Research Center, Department of Medicine, University of California San Francisco, San Francisco, United States of America; University of Texas Health Science Center at Houston, UNITED STATES

## Abstract

**Objectives:**

To investigate DKK-1 and SOST serum levels among patients with recent inflammatory back pain (IBP) fulfilling ASAS criteria for SpA and associated factors.

**Methods:**

The DESIR cohort is a prospective, multicenter French cohort of 708 patients with early IBP (duration >3 months and <3 years) suggestive of AxSpA. DKK-1 and SOST serum levels were assessed at baseline and were compared between the subgroup of patients fulfilling ASAS criteria for SpA (n = 486; 68.6%) and 80 healthy controls.

**Results:**

Mean SOST serum levels were lower in ASAS+ patients than healthy controls (49.21 ± 25.9 vs. 87.8 ± 26 pmol/L; p<0.0001). In multivariate analysis, age (p = 5.4 10^−9^), CRP level (p<0.0001) and serum DKK-1 level (p = 0.001) were associated with SOST level. Mean DKK-1 serum levels were higher in axial SpA patients than controls (30.03 ± 15.5 vs. 11.6 ± 4.2 pmol/L; p<0.0001). In multivariate analysis, DKK-1 serum levels were associated with male gender (p = 0.03), CRP level (p = 0.006), SOST serum level (p = 0.002) and presence of sacroiliitis on radiography (p = 0.05). Genetic association testing of 10 SNPs encompassing the *DKK-1* locus failed to demonstrate a significant contribution of genetics to control of DKK-1 serum levels.

**Conclusions:**

DKK-1 serum levels were increased and SOST levels were decreased among a large cohort of patients with early axial SpA compared to healthy controls. DKK-1 serum levels were mostly associated with biological inflammation and SOST serum levels.

## Introduction

Spondyloarthritis (SpA) is one of the most common inflammatory rheumatic diseases. The prevalence is estimated to be 0.5% to 3.4% [1,2]. In addition to the disabling rheumatic manifestations, some SpA patients develop severe extra-articular manifestations such as inflammatory bowel disease, uveitis or psoriasis. SpA is also characterized by the formation of syndesmophytes in the severe form of the disease. Treatment options are still limited to non-steroidal anti-inflammatory drugs (NSAIDs) as first-line therapy and biological treatment strategies that block specific immune mediators (e.g., tumor necrosis factor (TNF) blockers, and probably soon antibodies targeting interleukin 17A (IL-17A) or IL-23). Anti-TNF agents are commonly used in the refractory forms of the disease and have considerably improved the quality of life in patients by reducing clinical and biological disease activity. They also have significant efficacy in reducing subchondral-bone inflammatory lesions observed on axial MRI. Nevertheless, most previous studies have failed to demonstrate a structural benefit of TNF blockers in radiolographic disease progression as evaluated by the modified Stoke Ankylosing Spondylitis Spine Score after 2-year follow-up [3–6]. Conversely, Haroon et al. suggested that TNF blockers may reduce radiographic progression [7]. NSAIDs have been associated with reduced radiographic disease progression [8,9]. A better understanding of the pathogenic mechanisms involved in syndesmophyte formation is needed to develop targeted therapies for structural benefit and subsequent functional improvement in patients.

Secreted Wnt glycoproteins are among the major families of cell signaling molecules. Initially, they were shown to be involved in embryogenesis and tumorigenesis [10]. In recent years, several studies have implicated the Wnt canonical pathway in osteo-immunology and notably the bone formation process [11]. Wnt binding to its receptor complex, which includes low-density lipoprotein receptor-related protein 5/6 (LRP5/6) and Frizzled, initiates a number of intracellular signaling cascades leading to the accumulation of β-catenin in the cytoplasm and then to its translocation into the nucleus, where it enhances target gene expression. These genes are involved in osteoblastogenesis and the control of osteoclastogenesis.

Dickkopf-1 (DKK-1) and sclerostin (SOST) are two inhibitory proteins of the Wnt signalling pathway leading to osteoblastogenesis blockade. Both bind to LRP5/6 and block the Wnt/β-catenin canonical signalling pathway. Several murine models support their involvement in bone homeostasis. Osteopenia develops in mice transgenic for Dkk-1 [12] or SOST [13]. Conversely, mice with an inactivating mutation of DKK-1 show increased bone mass [14]. In humans, mutation of SOST leads to van Buchem disease, characterized by hyperosteosis [15].

In SpA, syndesmophyte development is secondary to endochondral formation (i.e., initial cartilage formation further replaced by bone) [16]. Therefore, DKK-1 and SOST may be involved in osteoblastogenesis dysregulation associated with syndesmophyte formation. The role of DKK-1 in the fusion of sacroiliac joints was revealed in human TNF transgenic mice [17]; DKK-1 blockade inhibited bone erosion of the sacroiliac joints and enhanced sacroiliac ankylosis, which strongly supports the potential role of Wnt signaling in the fusion of sacroiliac joints, the hallmark of SpA.

In addition, in mice, DKK-1 was found to induce SOST expression, which suggests complex cross-regulation between both proteins in bone homeostasis [18]. Moreover, both proteins bind the same LRP5/6 receptor and should mutually act as competitors in inhibiting the Wnt signaling pathway. Thus, additional investigation of both DKK-1 and SOST is needed to better define their roles in SpA.

Studies assessing serum level of DKK-1 in SpA patients are scarce and have generated conflicting results [19,20]. Discrepancies between published studies could be explained by the small number of patients studied, different methods of DKK-1 quantification, and lack of knowledge of DKK-1 serum levels in healthy individuals (e.g., the impact of age and gender on DKK-1 serum level). Robust data regarding DKK-1 serum levels among a large cohort of SpA patients and healthy controls are still lacking, as is our understanding of DKK-1 function in SpA.

We aimed to assess DKK-1 and SOST serum levels and associated factors in patients fulfilling the ASAS criteria for axial SpA within a large prospective cohort of patients with recent inflammatory back pain (IBP) (the cohort Devenir des Spondylarthropathies Indifferenciées Récentes [DESIR] [Outcome of Recent Undifferentiated Spondylarthropathies]). We also aimed to compare these levels with those in healthy controls to obtain more insight into the role of both Wnt inhibitors in SpA.

## Patients and Methods

### Patients and controls

This cross-sectional study quantified DKK-1 and SOST serum levels among all patients enrolled in the DESIR cohort and for whom data were available at baseline.

The DESIR cohort is a large national multicenter cohort developed to facilitate investigations of diagnostic and prognostic markers and etiologic, pathogenic and socio-economic factors among patients with early IBP suggestive of axial SpA. In fact, patients included in this cohort have IBP classified by the criteria of Calin et al. [21] or the Berlin criteria [22] (considering 2 of 4 items) of recent onset (> 3 months and < 3 years), with symptoms suggestive of SpA according to the local investigator’s assessment (score ≥ 5 on a 0–10 numerical rating scale, with 0, not suggestive of SpA, and 10, very suggestive). Patients included in DESIR cohort are planned to be followed up to 10 years. The main characteristics of the patients at baseline have been reported previously [23]. This cohort included 708 patients (mean age 33.8 ± 8.6 years, 46.2% men, and 57.3% positive for human leukocyte antigen B27 (HLA-B27)). The baseline characteristics included age, ethnicity, date at onset of IBP and peripheral arthritis, nature of IBP, presence of SpA features, relevant family history, and medication, including the use of NSAIDs and disease-modifying anti-rheumatic drugs (DMARDs). The duration of axial symptoms was defined as the time between the first axial symptom and the initial interview. As previously described [23], spinal mobility was measured by the Bath Ankylosing Spondylitis Metrology Index. Patients were asked to complete the Bath Ankylosing Spondylitis Disease Activity Index (BASDAI), Bath Ankylosing Spondylitis Functional Index (BASFI), Bath Ankylosing Spondylitis Global Index, Health Assessment Questionnaire, Medical Outcomes Survey Short Form 36, and Ankylosing Spondylitis Quality of Life questionnaire. Blood tests performed in the regional rheumatology centers tested for C-reactive protein (CRP) level, erythrocyte sedimentation rate (ESR), and HLA–B27 antigen as well as usual biologic parameters. High-sensitivity CRP (hs-CRP) was assessed as described [24]. The Ankylosing Spondylitis Disease Activity Score (ASDAS) [25] was calculated with CRP level. Radiographs were evaluated by 2 trained central readers blinded to any other data [26]. Radiographs of the sacroiliac joints were graded according to New York criteria. Lateral radiographs of the cervical and lumbar spine were used to calculate the modified Stoke Ankylosing Spondylitis Spine Score (mSASSS) [3]; an abnormal axial radiograph was defined with mSASSS ≥ 1. Data were extracted from the M0 DESIR database locked on June 30, 2010.

DKK-1 serum level was additionally assessed in 69 SpA patients from the SpondyloArthitis Caught Early (SPACE) cohort [27]. The SPACE cohort started in January 2009 and is an ongoing project. Patients ≥ 16 years old with chronic (almost daily) back pain for at least 3 months but <2 years, with onset before the age of 45 years, who were referred to the rheumatology outpatient clinic of Leiden University Medical Center (LUMC) were included after signing informed consent. The SPACE study protocol was approved by the LUMC medical ethics committee.

Controls were healthy subjects from the French Variété cohort. Variété is an open, prospective, French national, multicenter, non-randomized study of healthy volunteers established to determine normative data for insulin-like growth factor 1 (IGF-I) and other hormones in the general population (ClinicalTrials.gov Identifier: NCT01831648). The project aimed to establish normative data based on a large random selection from the general population, including representation from all age groups (about 100 subjects for each decade age range). Subjects with medical conditions and receiving medications that may affect IGF-I measurement were excluded. A total of 974 healthy subjects were recruited in 10 centers in France. Each subject underwent clinical examination. Personal medical history was recorded and gonadal status evaluated. Patients underwent biological standard workup, and 80 ml blood was sampled; serum and plasma was aliquoted and frozen and stored at -80°C before hormone measurements. All patients gave their informed consent to participate in the study, which was approved by the local ethics committee.

DKK-1 and SOST serum levels were assessed at baseline on the whole cohort, but case–control analyses and assessment of factors associated with increased DKK-1 serum level were restricted to the subgroup of patients fulfilling the ASAS criteria. DKK-1 and SOST serum levels at baseline were compared with those of 80 healthy controls from the Variété cohort. Because of no data in the literature on the impact of gender and age on DKK-1 serum level among the healthy population, 453 healthy controls from Variété cohort were further assessed for DKK-1 serum level in a broader age range than those matched for the DESIR cohort (18–79 years old, 47.5% females).

### Ethic statement

This study fulfills the current Good Clinical Practice Guidelines (French version) and received approval from the appropriate ethics committee. All patients gave their written informed consent. A website containing the detailed description of the centers, the organization of the cohort and the full detailed protocol and Case Record Form is at http://www.lacohortedesir.fr.

### DKK-1 locus genotyping

Single nucleotide polymorphisms (SNPs) encompassing the DKK-1 locus were genotyped to determine whether genetic variants of DKK-1 are associated with DKK-1 serum levels. Ten DKK-1 SNPs were chosen in order to cover the 74 Kb including DKK-1 locus with 5’ and 3’UTR regions of the gene. Seven out these 10 SNPs were previously studied in rheumatoid arthritis [28]. The 10 selected SNPs captured 66% of the *DKK-1* locus when considering SNPs with MAF higher than 0.10. SNPs were genotyped using a competitive allele-specific PCR system (KASpar genotyping, http://www.lgcgenomics.com). All genotyped SNPs had minor allele frequency (MAF) > 0.01 and were assessed for deviation from Hardy-Weinberg equilibrium. Of an initial 486 SpA patients fulfilling ASAS criteria, 58 patients were excluded from analysis based on self-reported non-Caucasian ancestry, and 2 individuals were excluded due to genotyping calling rate < 20%. Control individuals consisted by 1238 healthy individuals of Caucasian ancestry. Forty four control individuals were dropped from analyses based on individual genotyping calling rate < 20%. Thus, case-control analyses were performed based on comparisons of 426 SpA patients to 1,194 controls of Caucasian ancestry.

### Serum analyses

In the DESIR cohort, serum was prospectively collected from 2009 to 2010 at inclusion and stored in aliquots at -80°C in the Biological Resources Center at Bichat Hospital (accreditation AFNOR #34457). SOST and DKK-1 serum levels were assessed by sandwich ELISA (Biomedica Medizinprodukte, Vienna, Austria). ELISA tests involved an EVOLIS System (Bio-Rad, Hercules, CA, USA). DKK-1 serum samples were diluted 1:4 as recommended by the manufacturer for quantification. DKK-1 serum level from the DESIR cohort, the SPACE cohort and 80 age- and sex-matched healthy controls were assessed by the second-generation ELISA kit from Biomedica (Lot F112). DKK-1 serum level from 453 additional patients from the Variété cohort was assessed with the third-generation ELISA kit from Biomedica (Lot F125).

SOST serum level from the DESIR cohort and 80 age- and sex-matched controls was assessed with the first-generation ELISA kit from Biomedica (Lot Y113). SOST serum level was not assessed in a larger sample of healthy controls because data were available in the literature on impact of gender and age on levels [29,30].

For both DKK-1 and SOST, results are expressed in picomole per liter. For DKK-1, the conversion to picogram per milliliter is as follows: 1 pmol/L = 28.68 pg/mL.

Various quality controls were performed throughout the study: 2 internal controls were quantified on each ELISA plate for validation of each experiment. The first internal control (C1) was provided by the manufacturer and was an expected 3.1 to 5.9 pmol/L. All experiments were validated with a mean variation between all experiments of 4.38 (+/-0.42). The second internal control was a serum aliquot from a patient (C2) re-quantified on each used ELISA plate: C2 quantification varied from 24.06 to 33.06 pmol/L. Serum providing D.O. > 3.5 (>50 pmol/L) was diluted 1:2 and re-quantified. We used 80 serum samples tested in duplicate, which demonstrated no significant variation between both quantifications. Finally, we compared the DDK-1 ELISA (second-generation) test from Biomedica with the ELISA kit from R&D systems (human Dkk-1 DuoSet ELISA kit) and found a correlation between both tests (Spearman's rho (r_s_) = 0.72; p<0.0001, S1 Fig).

### Statistical analysis

Qualitative data are described as number (%) and quantitative data as mean (±SD) or median (interquartile range (IQR)) as appropriate. The Mann-Whitney test was used to compare independent samples. The correlation between serum levels and biochemical variables was evaluated by Spearman’s correlation coefficient (r_s_). Variables included in univariate analysis were weight, body mass index (BMI; kg/m^2^), disease duration, erythrocyte sedimentation rate (ESR; mm/h), C-reactive protein level (CRP; mg/L), BASFI, BASDAI, serum calcium or phosphate level, and lumbar-spine and total-femur bone mineral density. Variables identified as significantly associated with DKK-1 or SOST levels on univariate analysis (at p = 0.10) were entered into non-parametric linear regression models. DKK-1 serum levels are normally distributed and were studied as a continuous or a dichotomous variable (patients with high levels of DKK-1 [3^rd^ and 4^th^ quartiles] (DKK-1>36 pmol/L) compared to patients with low levels [1^st^ and 2^nd^ quartiles]) (DKK-1 ≤ 36 pmol/L)) in multivariate analyses (linear regression and logistic regression, respectively) to account for covariates associated with DKK-1 serum levels such as CRP, SOST serum level and presence of sacroiliitis on radiography. *P*<0.05 was considered statistically significant. Statistical analyses involved use of R 3.1.0 (R Core Team [2014], R Foundation for Statistical Computing, Vienna, Austria. http://www.R-project.org/).

Genetic association analyses were performed to determine whether individual SNPs were associated with disease/phenotype/DKK-1 serum levels using the STATA program (v.12; College station, Texas). The contribution of the 10 SNPs was assessed according to a recessive, dominant or additive model of transmission in uni- and multivariate analyses. For SNPs that were in linkage disequilibrium (D’>0.95 and r^2^>0.65), haplotypes were estimated using PLINK and haplotype association analyses (bivariate) were performed using Haploview.

## Results

### Patients with early SpA and controls

In total, 708 patients have been included in the DESIR cohort (46.2% male). The mean age was 33.8 ±8.6 years and the mean duration from the onset of symptoms to referral to the rheumatologist was 18.8±11.6 months, corresponding to patients with early IBP suggestive of SpA. Overall, 486 patients fulfilled the ASAS criteria for axial SpA (mean age 32.5 ± 8.6 years, 50.2% men, and 83.7% HLA–B27 positive). Among these patients, 80% were exposed to NSAIDs at baseline. Characteristics of disease activity and disease severity are in Table 1. The 80 healthy controls (51% men, mean age 32 ±9.1 years) were age- and gender-matched with axial SpA patients from the DESIR cohort. The age range of the 453 healthy controls from the Variété cohort (238 males) was 18 to 79 years.

**Table 1:** Baseline demographics and disease characteristics of Assessment of Spondyloarthritis International Society (ASAS+) patients from the DESIR cohort.

	No. with available data	ASAS+ patients (n = 486)
Gender (male %)	486	50.2
Age (years)	486	32.5±8.6
Disease duration (months)	479	18.8±11.6
HLA-B27+ (%)	485	83.7
CRP level (mg/dl)	469	9.3±13.9
hs-CRP level (mg/dl)	470	8.1±14.2
ESR (mm)	468	14.8±16.8
BASDAI	475	43±20.4
BASFI	475	29.7±22.4
BASMI	465	2.2±0.9
Radiological sacroiliitis (%)	476	27.3
mSASSS ≥ 1 unit (%)	460	13.2
Current use of oral NSAIDs (%)	396	80
DKK-1 level (pmol/L)	479	30.3±15.5
SOST level (pmol/L)	478	49.2±26.1

Data are mean±SD unless indicated

HLA-B27, human leukocyte antigen B27; CRP, C-reactive protein; hs-CRP, high-sensitivity CRP; ESR, erythrocyte sedimentation rate; BASDI, Bath Ankylosing Spondylitis Disease Activity Index; BASFI, Bath Ankylosing Spondylitis Functional Index; BASMI, Bath Ankylosing Spondylitis Metrology Index; mSASSS, modified Stoke Ankylosing Spondylitis Spine Score; NSAIDs, nonsteroidal anti-inflammatory drugs; DKK-1, Dickkopf-1; SOST, sclerostin

### Decreased SOST serum level among patients with early SpA

**Fig 1:**
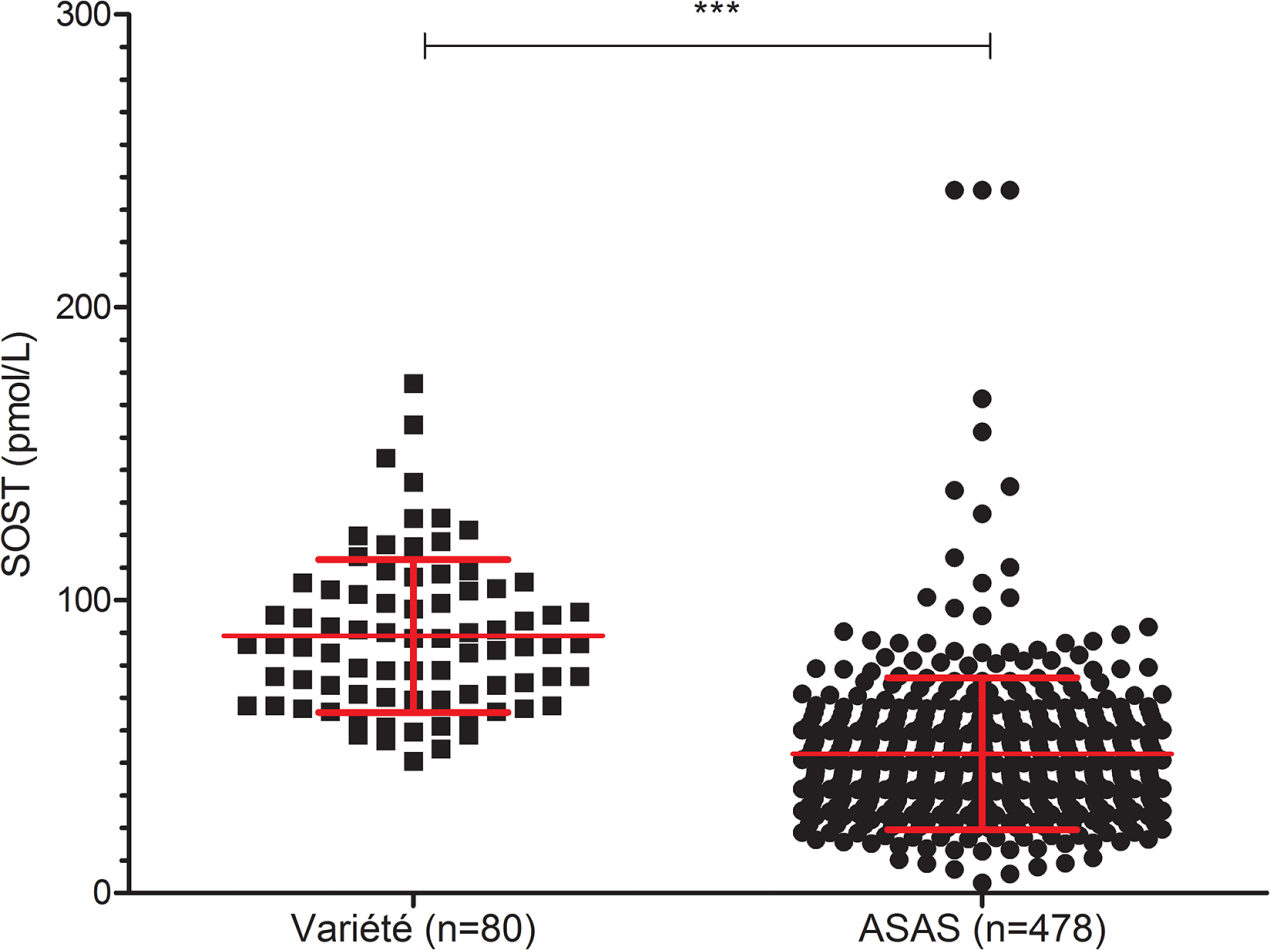
Serum sclerotin (SOST) level among patients with axial spondyloarthritis (SpA) and controls at baseline. Each point represents 1 patient. Data are mean ± SD. ***p<0.0001.

**Fig 2:**
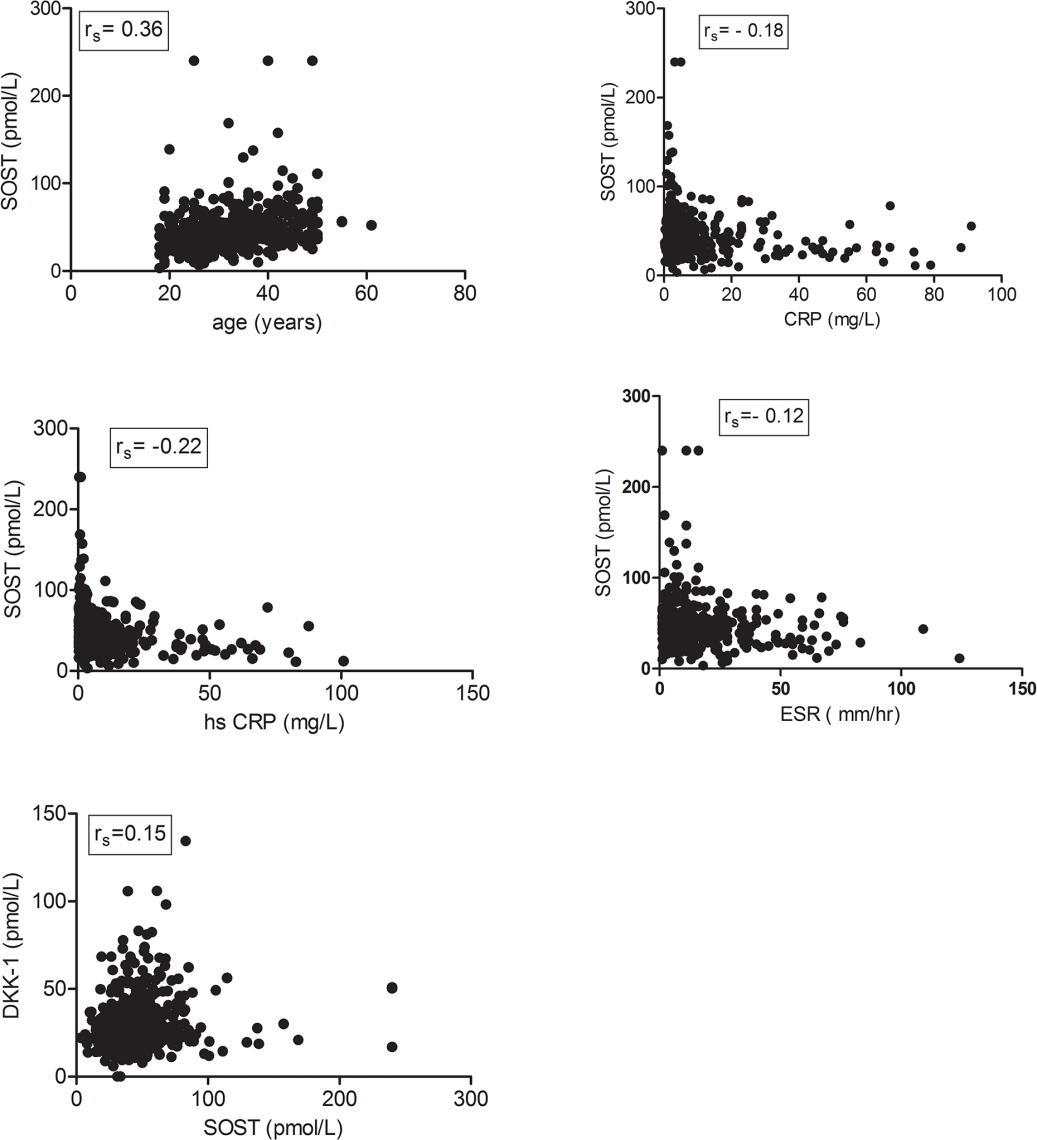
Correlation of SOST serum level with age. (A) (r_s_ = 0.36; p<0.0001), C-reactive protein (CRP) level (B) (r_s_ = -0.18; p = 0.0001), high-sensitivity CRP (hs-CRP) level (C) (r_s_ = -0.22; p<0.0001), erythrocyte sedimentation rate (ESR) (D) (r_s_ = -0.12, p = 0.007) and Dickkopf-1 (DKK-1) serum levels (E) (r_s_ = 0.15, p = 0.0008); r_s_: Spearman correlation coefficient.

SOST serum level was significantly lower in axial SpA patients than in controls from the Variété cohort (mean 49.21 ± 25.9 vs. 87.8 ± 26 pmol/L; p<0.0001) (Fig 1). SOST serum level was significantly correlated with age (r_s_ = 0.36; p = 2.2 10^−16^), CRP level (r_s_ = -0.18; p = 0.0001), hs-CRP level (r_s_ = -0.22; p = 10^−6^), and ESR (r_s_ = -0.12, p = 0.007) (Fig 2A–2D) (Table 2). SOST serum level in axial SpA patients was lower for those with than without sacroiliitis on radiography (n = 130 vs. n = 346; mean 42.95 ± 18.4 vs 49.25 ± 28.91 pmol/L; p = 0.023). SOST serum level did not differ between patients with than without axial structural lesions (mSASSS ≥ 1 unit vs 0).

**Table 2:** Correlation between SOST serum level and characteristics of ASAS+ patients of the DESIR cohort.

Characteristic	N	Spearman r_s_	p-value^a^	β-coefficient	p-value^b^
DKK-1 level	479	0.15	0.0008	0.25	0.001
hs-CRP level	477	-0.22	10^−6^		
CRP level	462	-0.18	0.0001	-0.29	0.0008
ESR	461	-0.12	0.007		
Age	479	0.36	<2.2 10^−16^	0.83	< .0001

^a^: Univariate analysis

^b^: Multivariate analysis with hs-CRP and ESR excluded

A correlation between SOST and DKK-1 serum levels was observed (r_s_ = 0.15, p = 0.0008) (Fig 2E) as previously described [31]. Nevertheless, such correlation was weak when assessed on the whole population of SpA patients. This correlation was higher in the subgroup of patients with increased levels of DKK-1 (DKK-1/SOST ratio > 1) corresponding to a third of ASAS positive patients (rs = 0.83; p<0.0001).

On multivariate analysis, age (p = 5.4 10^−9^), CRP level (p<0.0001) and DKK-1 serum level (p = 0.001) were associated with SOST level (Table 2).

### Increased serum DKK-1 level in SpA patients

**Fig 3:**
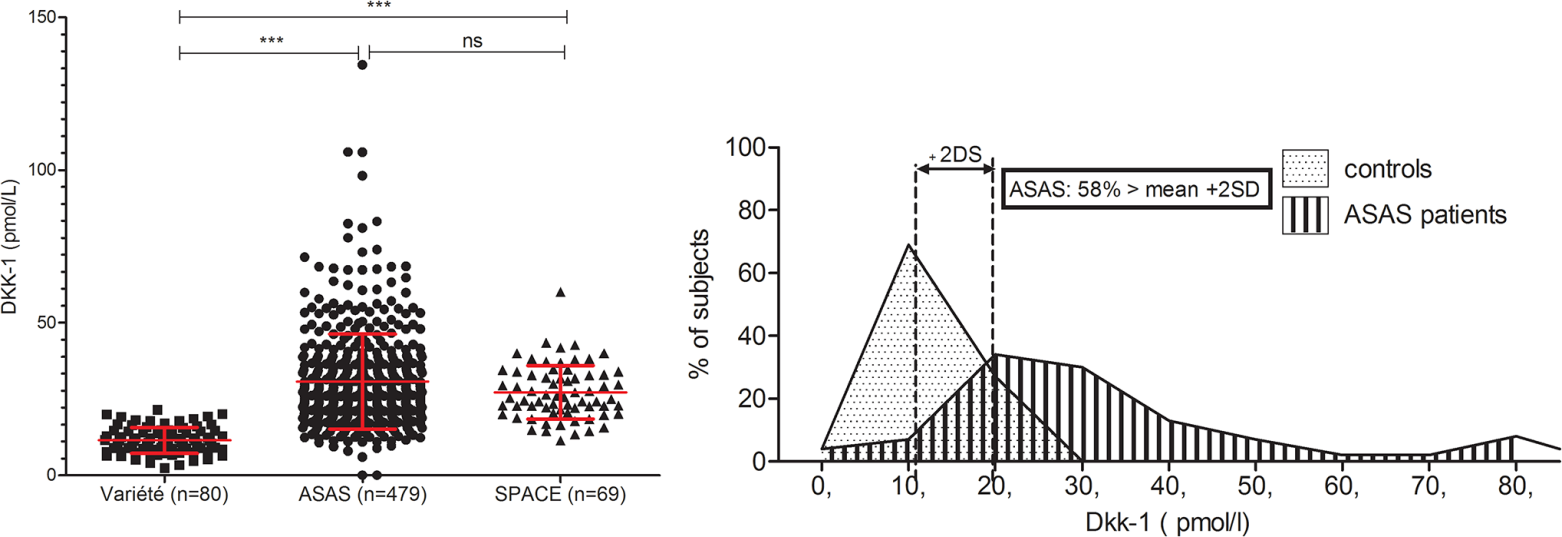
(A) Serum DKK-1 level among axial SpA patients and controls at baseline. Each point represents 1 patient. Data are mean ± SD. *** p< 0.0001. (B) Distribution of DKK-1 levels in controls and axial SpA patients.

**Fig 4:**
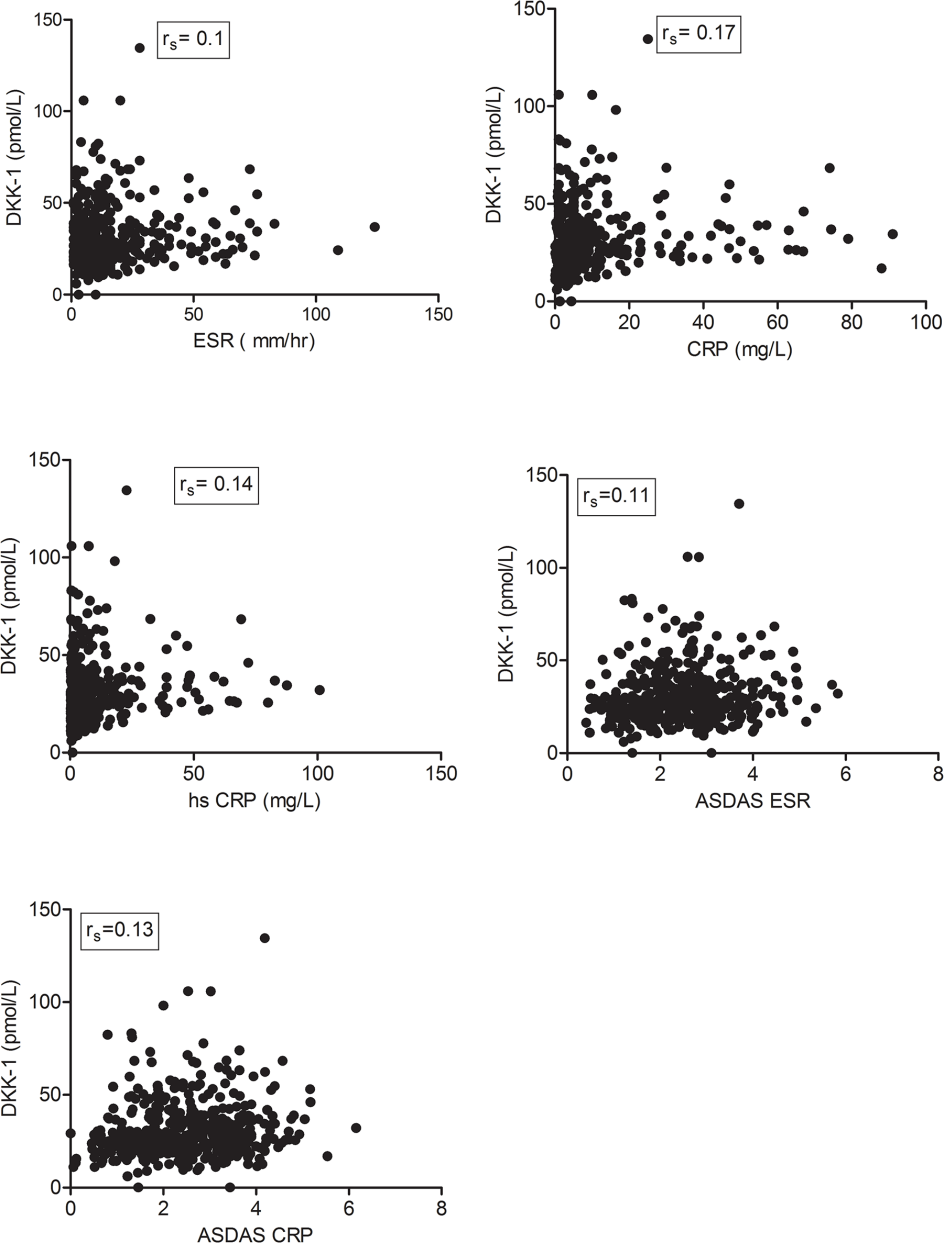
Correlation of DKK-1 serum level with systemic inflammation assessed by ESR. (A) (r_s_ = 0.1, p = 0.03), CRP level (B) (r_s_ = 0.17; p = 0.0001), hs-CRP level (C) (r_s_ = 0.14; p = 0.003), Ankylosing Spondylitis Disease Activity Score (ASDAS)-ESR (D) (r_s_ = 0.11; p = 0.02) and ASDAS-CRP level (E) (r_s_ = 0.13; p = 0.004); r_s_: Spearman correlation coefficient.

DKK-1 serum level was significantly higher in axial SpA patients than controls (mean 30.03 ± 15.5 vs. 11.6 ± 4.2 pmol/L; p<0.0001) (Fig 3A), with almost no overlap between patients and controls (Fig 3B). This finding was confirmed in an independent SpA cohort (SPACE; Fig 3A). DKK-1 serum level was weakly significantly correlated with systemic inflammation assessed by ESR (r_s_ = 0.1, p = 0.03), CRP level (r_s_ = 0.17; p = 0.0001), hs-CRP level (r_s_ = 0.14; p = 0.003), ASDAS-ESR (r_s_ = 0.11; p = 0.02) and ASDAS-CRP level (r_s_ = 0.13; p = 0.004). (Fig 4A–4E) (Table 3) but not disease activity assessed by the BASDAI (r_s_ = 0.052; p = 0.26). The association of DKK-1 serum level and ASDAS-ESR may be related to systemic inflammation rather than patient-reported disease activity.

**Table 3:** Correlation between DKK-1 serum level and characteristics of ASAS+ patients of the DESIR cohort.

Characteristic	N	Spearman r_s_	p-value^a^	β-coefficient	p-value^b^
SOST level	475	0.15	0.0008	0.088	0.002
ASDAS-ESR	456	0.11	0.02		
ASDAS-CRP	394	0.13	0.004	1.54	0.03
hs-CRP level	477	0.14	0.003		
CRP level	462	0.17	0.0001	0.15	0.006
ESR	461	0.1	0.03		
Gender (male)	486	NA	0.08	-3.18	0.03
Sacro-iliitis	476	NA	0.05	3.37	0.05
HLA-B27	485	NA	0.04	2.42	0.2

ASDAS, Ankylosing Spondylitis Disease Activity Score

^a^: Univariate analysis

^b^: Multivariate analysis with hs-CRP, ESR, and ASDAS-ESR excluded

DKK-1 serum level was significantly higher in HLA-B27-negative than-positive patients (n = 79 vs n = 406; mean 33.97 ± 19.39 vs 29.99 ± 14.56 pmol/L; p = 0.04). DKK-1 serum level was associated but not significantly with sacroiliitis on radiography (mean 33.02 ± 16.47 vs 29.93 ± 15.28, p = 0.056). None of the other studied variables (age, gender, weight, BASDAI, NSAIDs, corticosteroids or DMARDs intake) were significantly correlated with DKK-1 serum level.

DKK-1 serum level was increased but not significantly in patients with compared to without axial involvement (mSASSS ≥ 1 unit vs 0; n = 61 vs. n = 399; mean 33.42 ± 17.11 vs 30.66 ± 15.53 pmol/L; p = 0.21).

Multivariate analysis revealed a significant positive association of DKK-1 serum level and female gender (p = 0.03), CRP level (p = 0.006), SOST serum level (p = 0.002) and the presence of sacroiliitis on radiography (p = 0.05) (Table 3).

### Study of DKK-1 polymorphisms in relation to structural damage at baseline and DKK-1 serum levels

Univariate analyses revealed a borderline significant association between rs7083441 and rs11001445 with the presence of syndesmophytes at baseline (P_trend_ = 0.08 and P_trend_ = 0.07, respectively). However, multivariate analyses including variables previously associated with structural damage at baseline (CRP, gender, smoking) failed to demonstrate an association of these SNPs (or any of the 8 other genotyped SNPs) with structural damage at baseline (data not shown). None of the studied polymorphisms contributed significantly to DKK-1 serum levels, regardless of the genetic model assumed (recessive, dominant, additive), in either univariate or multivariate analyses (data not shown). Haplotype analyses also did not reveal evidence of association with DKK-1 serum levels.

## Discussion

On investigating the serum levels of DKK-1 and SOST in a large cohort of patients with recent axial SpA, we have demonstrated increased total DKK-1 level and decreased SOST level among patients as compared with controls. Of importance, quantifications were not biased by DMARDs and or anti-TNF treatments because all patients included in the cohort were naïve of these drugs at baseline. Decreased SOST level in SpA patients was previously described [32] and is expected in a disease associated with new bone formation. Conversely, results for DKK-1 are new.

We found a significant association of low SOST serum level and sacroiliitis seen on radiography (structural damage) among SpA patients from the DESIR cohort. Appel et al. also reported low serum level of SOST in SpA patients significantly associated with the formation of new syndesmophytes [32], and SOST inhibition (associated with TNF inhibition) led to a significant regression of cortical bone erosions in TNF transgenic mice [33]. Subchondral inflammation, bone erosion and exuberant bone formation being a continuous process in SpA, low level of SOST at baseline could be associated with new bone formation resulting from overwhelming healing occurring after inflammation and bone erosion.

Controls were age- and sex-matched with patients. To our knowledge, our work provides new data based on a large cohort concerning the variation in DKK-1 level by age and gender in healthy controls. DKK-1 serum level was not severely affected by these demographic characteristics. Conversely, age was a significant predictor of SOST serum level in SpA patients. The correlation between age and SOST level has not been reported in SpA but has been reported among healthy women [30].

DKK-1 serum level was greatly elevated in SpA patients, without almost no overlap between data for patients and controls. We previously demonstrated increased DKK-1 level in the French cohort ESPOIR of rheumatoid arthritis and associated with increased risk of radiographic progression [34]. Reconciling both results is difficult. In fact, as a marker of local bone resorption, increased DKK-1 level is somewhat expected in RA but is unexpected in SpA, with bone formation the hallmark of the disease. This increase may be linked to erosive lesions. Unfortunately, we cannot answer this question because patients exclusively presenting erosive lesions are underrepresented in the DESIR cohort. Prospective follow-up will help differentiate erosive from sclerosing lesions. Nevertheless, the distribution of DKK-1 serum level among SpA patients poorly supports this hypothesis because increased DKK-1 level largely represented SpA patients, more so than patients with exclusive erosive lesions.

Diarra et al. previously reported decreased serum DKK-1 level in SpA patients [20], but Daoussis et al. reported higher serum DKK-1 level among SpA patients than controls [19]. These results are not contradictory because the ELISA test used in each study differed: in the study from Diarra et al., DKK-1 serum level was assessed with human LRP6-coated plates (also named functional quantification of DKK-1), whereas Daoussis et al. quantified circulating DKK-1 level with a classical sandwich ELISA. Therefore, these latter results agree with our study assessing free DKK-1 serum level. Nevertheless, the study by Daoussis et al. relied on a small sample of patients (n = 45) and assessed DKK-1 serum level among patients with overt ankylosing spondylitis fulfilling the New York diagnostic criteria. The results obtained in DESIR cohort involving SpA patients with a short disease duration (18.8 ±11.6 months) are thus complementary, showing that increased serum level of free DKK-1 is not restricted to the overt severe structural forms of the disease but should be a more long-standing process. Daoussis et al. also studied the functional consequence of increased circulating DKK-1 level in SpA patients. The authors assessed the effect of sera from SpA patients and controls on Wnt pathway activation. Jurkat T cells were treated with LICL, a known activator of the Wnt signalling pathway, then incubated with sera from SpA patients or controls and Wnt pathway activation was assessed by measuring the level of dephosphorylated β-catenin (the active form). Serum from SpA patients was unable to inhibit Wnt signalling pathway as compared with control serum, despite increased level of circulating DKK-1.

Therefore, in SpA patients, free DKK-1 level is increased, but functional DKK-1 seems to be decreased. The missing link between these observations could be abnormal binding of DKK-1 to its receptor among SpA patients. The origin of this dysfunction is unclear. DKK-1 and not its receptor LRP6 may be dysfunctional because results observed for SOST, which shares the same receptor, were opposite in our study. Second, based on our results genetic variation appears to be unlikely to explain the increased DKK-1 serum levels. Further, neither linkage nor genome-wide association studies have demonstrated a linkage or an association between the DKK-1 locus on chromosome 10 and SpA [35]. Cortes et al previously assessed the role of several polymorphisms of DKK-1 on SpA structural severity but failed to demonstrate evidence of association, although only 3 DKK-1 SNPs were studied [36]. Our study, which assessed 10 SNPs encompassing *DKK-1* locus failed to provide evidence of genetic association with DKK-1 serum levels and/or with structural damage at baseline. However, it is possible that rare coding variants might interfere with DKK-1 function for a small subset of SpA patients. Alternatively, post-translational modifications such as glycosylation or phosphorylation might lead to abnormal binding of DKK-1 on LRP5/6.

The variables most significantly associated with DKK-1 serum level were SOST serum level and those linked to biological inflammation, which agrees with the induction of DKK-1 by TNF [18]. Moreover, TNF induces SOST in mature osteoblasts and is primarily mediated by DKK-1 [18]. However, unlike RA, SpA is not characterized by high systemic inflammation. Thus, inflammation should not explain alone the increased serum level of DKK-1.

Univariate analyses revealed high DKK-1 level (P_trend_ = 0.056) and low SOST level (P = 0.02) among patients with sacroiliitis on radiography. As well, DKK-1 serum level was significantly reduced among HLA-B27–positive patients. In fact, these patients are expected to have fewer structural or inflammatory lesions on radiography, thus fulfilling the “clinical arm” of the ASAS criteria. Thus, DKK-1 level is increased when SOST level is decreased among patients with structural lesions seen on radiography. DKK-1 might be unable to bind LRP5/6 correctly among some SpA patients, as discussed previously. DKK-1 and SOST may compete for binding at LRP5/6, assuming that a higher affinity of SOST for its receptor would lead to increased levels of free DKK-1. Nevertheless, the positive and significant correlation between DKK-1 and SOST does not support this latter hypothesis, at least among one third of the SpA patients corresponding to those with a DKK-1/SOST ratio >1.

In conclusion, we demonstrate higher total serum DKK-1 levels but lower serum levels of SOST in SpA patients compared to controls. We also demonstrate an association between DKK-1 and SOST levels and systemic inflammation and between SOST levels and age among SpA patients. Our results suggest that increased DKK-1 serum levels among SpA patients is unlikely to be explained by genetic variation at that locus. Prospective follow-up will help improve our knowledge of the role of Wnt/DKK-1/SOST pathways in SpA. First it will help clarify the interaction between treatment (NSAIDs, TNF-blockers) and DKK-1 or SOST levels; Second, it will help better delineate the role of DKK-1 and SOST in structural disease progression (i.e., syndesmophyte formation) and/or in systemic bone loss in SpA. Finally, these results raise the question of a potential dysfunction of DKK-1 linked with post-transcriptional modifications. Further studies are needed to unravel this puzzle to open up new therapeutic perspectives.

## Supporting Information

S1 Fig Correlation of DKK-1 serum level assessment between 2 different ELISA kits (R&D and Biomedica). (TIF)
