# Supplementary figures and images for: Increase in Dickkopf-1 Serum Level in Recent Spondyloarthritis. Data from the DESIR Cohort
Source: PLoS One. 2015 Aug 27;10(8):e0134974. doi: 10.1371/journal.pone.0134974 (PMC4552086; doi:10.1371/journal.pone.0134974)

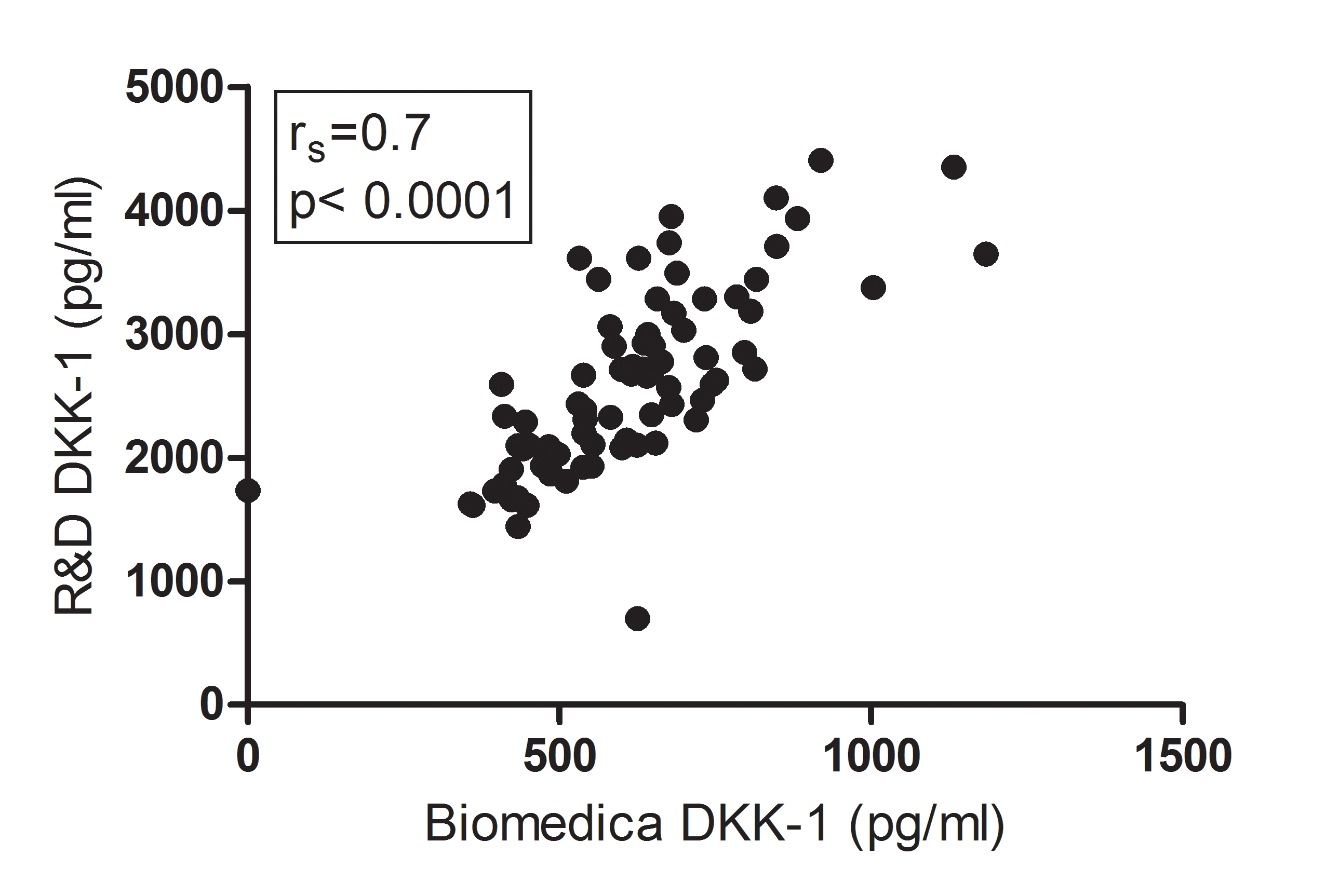

Supplement: S1 Fig — (TIF) [file pone.0134974.s001.tif]
